# Effect of the Height of a 3D-Printed Model on the Force Transmission and Thickness of Thermoformed Orthodontic Aligners

**DOI:** 10.3390/ma17123019

**Published:** 2024-06-20

**Authors:** Omar Ghoraba, Christoph Bourauel, Mostafa Aldesoki, Lamia Singer, Ahmed M. Ismail, Hanaa Elattar, Abdulaziz Alhotan, Tarek M. Elshazly

**Affiliations:** 1 Oral Technology, Dental School, University Hospital Bonn, 53111 Bonn, Germany; 2 Orthodontic Department, Dental School, University Hospital Bonn, 53111 Bonn, Germany; 3 Biomaterials Department, Faculty of Dentistry, Ain Shams University, Cairo 11566, Egypt; 4 Orthodontic Department, Faculty of Dentistry, Suez Canal University, Ismailia 8366004, Egypt; 5 Department of Dental Health, College of Applied Medical Sciences, King Saud University, P.O. Box 10219, Riyadh 12372, Saudi Arabia

**Keywords:** biomechanics, removable thermoplastic appliance, thermoforming, 3D printing, micro-CT

## Abstract

This research aims to investigate the influence of model height employed in the deep drawing of orthodontic aligner sheets on force transmission and aligner thickness. Forty aligner sheets (Zendura FLX) were thermoformed over four models of varying heights (15, 20, 25, and 30 mm). Normal contact force generated on the facial surface of the upper right central incisor (Tooth 11) was measured using pressure-sensitive films. Aligner thickness around Tooth 11 was measured at five points. A digital caliper and a micro-computed tomography (µ-CT) were employed for thickness measurements. The normal contact force exhibited an uneven distribution across the facial surface of Tooth 11. Model 15 displayed the highest force (88.9 ± 23.2 N), while Model 30 exhibited the lowest (45.7 ± 15.8 N). The force distribution was more favorable for bodily movement with Model 15. Thickness measurements revealed substantial thinning of the aligner after thermoforming. This thinning was most pronounced at the incisal edge (50% of the original thickness) and least at the gingivo-facial part (85%). Additionally, there was a progressive reduction in aligner thickness with increasing model height, which was most significant on the facial tooth surfaces. We conclude that the thermoplastic aligner sheets undergo substantial thinning during the thermoforming process, which becomes more pronounced as the height of the model increases. As a result, there is a decrease in both overall and localized force transmission, which could lead to increased tipping by the aligner and a diminished ability to achieve bodily movement.

## 1. Introduction

Orthodontic clear aligners offer superior aesthetics and improved hygiene compared to bracket systems. They also demand less chair time and fewer clinical visits, making them more convenient for clinicians. As a result, there is significant current demand for aligners, leading to their widespread use in the dental market [1,2]. However, attaining optimal clinical outcomes with aligners relies on optimizing various factors [3,4], including material type [5], thickness [6], edge shape, extension [7], and the degree of activation [8]. Therefore, enhancing the biomechanical understanding of applied forces is crucial for improving the safety and predictability of aligner treatments, especially considering observed discrepancies between planned and actual outcomes, which may result from inaccuracies in predicting forces applied to teeth [4]. Furthermore, it is imperative to carefully consider each single factor during the manufacturing and application of aligners, based on well-established scientific evidence. This approach will lead to the development of a standardized method for practitioners and companies, ensuring the best treatment outcomes. Additionally, standardizing all studies in the literature will facilitate the comparison of similar research endeavors.

The predominant method of aligner manufacturing in the industry involves deep drawing of a thermoplastic sheet onto a 3D-printed model. This thermoforming process has been noted to significantly influence material properties [9] and may lead to the thinning of sheets over the model [10]. A direct relationship has been identified between aligner thickness and force generation [8,11]. Various research methods have been employed to assess the mechanics of orthodontic aligners. Pressure-sensitive films have gained broad acceptance in the literature and have been used in numerous studies to quantify contact force between aligners and teeth [10,12,13,14]. Moreover, in several studies [15,16], micro-computed tomography (µ-CT) was utilized to achieve precise measurements of aligner thickness.

To date, there is a lack of existing studies documenting the effect of 3D-printed model height, used in deep drawing of orthodontic aligner sheets, on the thermoformed aligner thickness and the force transmitted to the misaligned tooth. Therefore, the originality of the present study lies in addressing this gap through the utilization of pressure-sensitive films, a digital caliper, and µ-CT imaging, and establishing a link among these methods.

## 2. Materials and Methods

**Figure 1:**
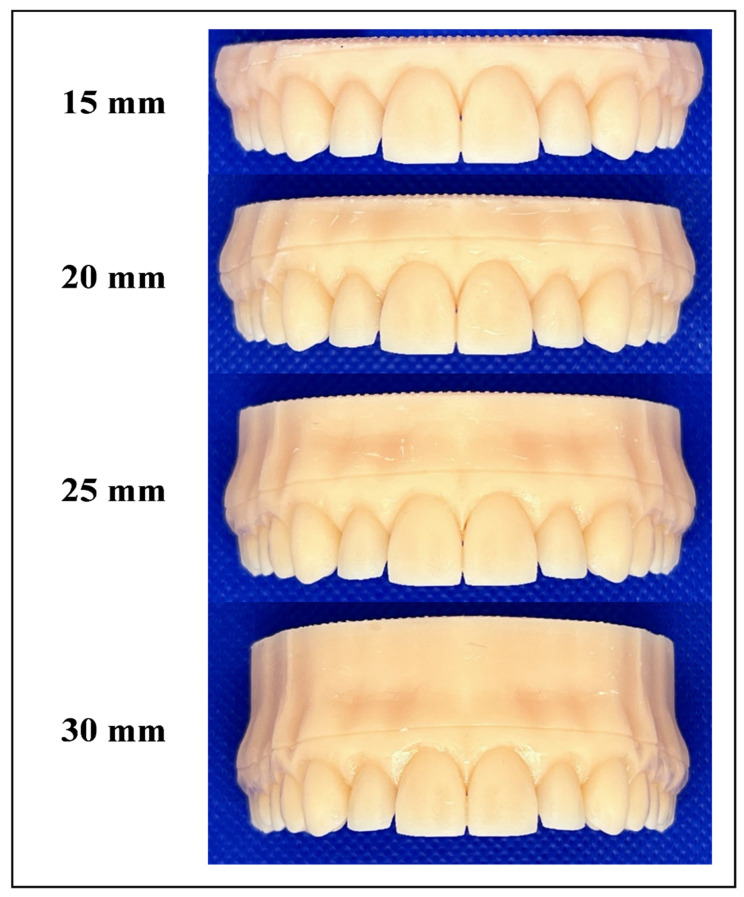
Different 3D-printed aligned resin models used for thermoforming, exhibiting varying heights. A shallow groove was positioned 2 mm beyond the gingival line to facilitate the standardization of aligner trimming.

**Figure 2:**
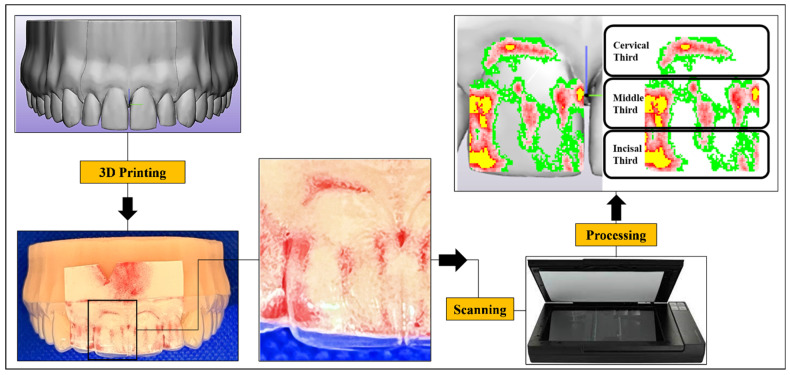
A visual representation of the procedure followed for measuring the local normal contact force, generated on the crown of a maxillary right central incisor (Tooth 11), using Fuji^®^ pressure-sensitive film. The pressurized film underwent scanning with a scanner, and the 2D scan was superimposed over a 3D digital model of the tooth to enhance visualization.

A digital model of an upper full-dentate jaw (Digimation Corp., St. Rose, LA, USA) was imported into image processing software (3-matic 17.0; Materialise, Leuven, Belgium). This model served as the basis for designing five distinct models. Four were with aligned teeth, designed for thermoforming purposes, but each had a different height from the occlusal table to the model base, specifically 15, 20, 25, and 30 mm (Figure 1). The fifth model, designed for force measurement purposes, intentionally introduced misalignment in the upper right central incisor (Tooth 11), moving it to be bodily translated by 0.2 mm toward the facial side. In this misaligned model, a 100 µm space was incorporated around the four incisors and their corresponding gingival area (housing space) to eliminate the influence of the inherent thickness of the pressure-sensitive films used for force measurements (Figure 2). All five digital models were exported as STL files to a digital light processing (DLP) 3D printer (P20+; Straumann, Basel, Switzerland) and printed using dental model resin (P pro resin; Straumann, Basel, Switzerland).

Forty aligner sheets made from 0.76 mm thick multilayered thermoplastic sheets (Zendura FLX; Bay Materials LLC, Fremont, CA, USA) were deep-drawn over the four models (*n* = 10) using a thermoforming device (Biostar; Scheu-Dental GmbH, Iserlohn, Germany), and following the manufacturer’s guidelines (Code 162; heating for 50 s at 220 °C and pressure forming at 4.8 bar, then cooling for 60 s). All aligners were trimmed by a single trained investigator into a straight 2 mm extended design, guided by a shallow pre-designed groove in the model (Figure 1).

The local normal contact force transmitted by each aligner to the crown of Tooth 11 was recorded using pressure-sensitive films (Fuji^®^ Prescale Film; Fuji Film, Tokyo, Japan), following parameters and steps detailed in a recent study [10]. These films are available as either mono-sheet or two-sheet types, each provided at various sensitivity levels with specific operational ranges. In this study, we utilized the low-pressure (LW) two-sheet films, which consist of an A-transfer sheet and a C-developer sheet. The combined thickness of these sheets is approximately 100 µm, as reported by the manufacturer. The A-film contains microcapsules designed to rupture under applied pressure, reacting with an active developing layer on the C-film. This reaction causes a change in color intensity from pale pink to dark red, corresponding to the pressure intensity.

These films were cautiously seated in the pre-designed housing space in the misaligned model, and then the aligner was inserted carefully to avoid undesirable pressure. For each measurement, the pressure film was positioned between the aligner and the printed model for 2 min to measure continuous pressure, following the manufacturer’s guidelines. Subsequently, each pressurized film was scanned using a specialized scanner (EPSON Perfection V300 series; SEIKO Epson CORPORATION, Tokyo, Japan) with a maximum resolution of 12,800 dpi and a scanning resolution of 200 dpi. Digitalization and quantification of force were performed using specialized software (FPD-8010E analysis system; Fuji^®^ Film, Tokyo, Japan) (Figure 2). This software allows the direct calculation of force, by multiplying the average pressure (MPa) by the pressurized area (mm^2^) (as per the software manual):

Force = Pressure × area

Force values (N) at the incisal (I), middle (M), and cervical (C) thirds of the facial surface of Tooth 11, along with the overall force (N) across the facial surface of the tooth, were recorded (Figure 2).

Before scanning, the scanner was calibrated using a special sheet provided by the manufacturer. The type of pressurized film was selected accordingly (here, we selected LW). Room temperature and relative humidity were monitored throughout the testing procedure using a digital meter (Technoline WS 9440 Indoor Climate Station; Technotrade GmbH, Wildau, Germany), and these values were input into the software for accurate measurement and calculation, as the rupture of microcapsules and the resulting color density depend on both temperature and humidity. The temperature was maintained at 21 ± 2 °C, and relative humidity at 29 ± 4%. During scanning, the lustrous side of the pressurized A-Transfer sheet was placed facing down, with the rough side (active color generation side) facing up, as per the manufacturer’s instructions.

To eliminate any effect of lag time between testing and scanning, a sensitivity analysis was conducted by scanning the same pressurized films at three different intervals over one hour (immediately, after 30 min, and after an hour). No significant difference in recorded pressure values was found between different scanning times when all other factors were kept constant.

**Figure 3:**
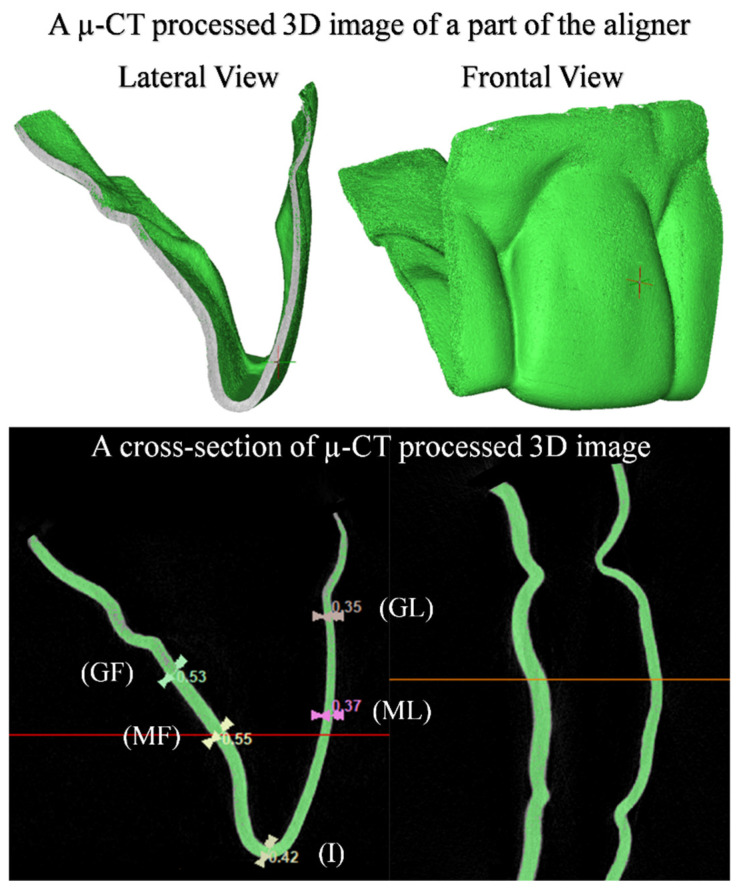
A µ-CT processed 3D image for a part of the aligner over the upper right central incisor (Tooth 11) for measurement of aligner thickness by MIMICS 25.0 software, at various reference points (gingivo-facial (GF), mid-facial (MF), incisal (I), mid-lingual (ML), gingivo-lingual (GL)).

Moreover, the thickness of the aligners was measured at various reference points (gingivo-facial (GF), mid-facial (MF), incisal (I), mid-lingual (ML), gingivo-lingual (GL)) along the midline of the aligner part covering the crown of Tooth 11. Two measurement methods were employed to enhance accuracy, ensure validation, and provide a means of cross-verification. The first method used a digital caliper (Dial Caliper D; Aura-Dental GmbH, Aura an der Saale, Germany), while the second method employed µ-CT (Skyscan 1174; Skyscan, Kontich, Belgium), with a resolution of 22 μm, an accelerating voltage of 50 kVp, a beam current of 800 μA, an exposure time of 22 s, a rotation of 360° at a 0.4° angular step, and without using filters. The mean total number of slices was 1018, and the average scanning time was one and a half hours. The 2D data were transferred as DICOM files into a 3D interactive medical image processing system (Mimics 25.0; Materialise, Leuven, Belgium). Mimics provided advanced visualization and segmentation capabilities through image radio-opacity thresholding. An automated process within Mimics allowed the generation of a 3D model for each specimen by expanding a threshold region across the complete stack of scans (Figure 3). Identifying the optimal radio-opacity threshold was a crucial and highly sensitive step in determining and analyzing the 3D objects.

All measurements were carried out by a single well-trained operator. Additionally, caution was always taken into consideration during the experiment to ensure consistency and standardization.

## 3. Statistical Analysis

Normality was assessed by examining the data distribution and conducting the Shapiro–Wilk test. The data exhibited a parametric distribution and are presented as mean values with corresponding standard deviations. Statistical analysis was conducted using a one-way ANOVA, followed by a post hoc Tukey HSD Test. The significance level (α) was set at *p* ≤ 0.05. The statistical analysis was carried out using IBM SPSS Statistics version 25 for Windows (IBM, Endicott, NY, USA).

## 4. Results

**Figure 4:**
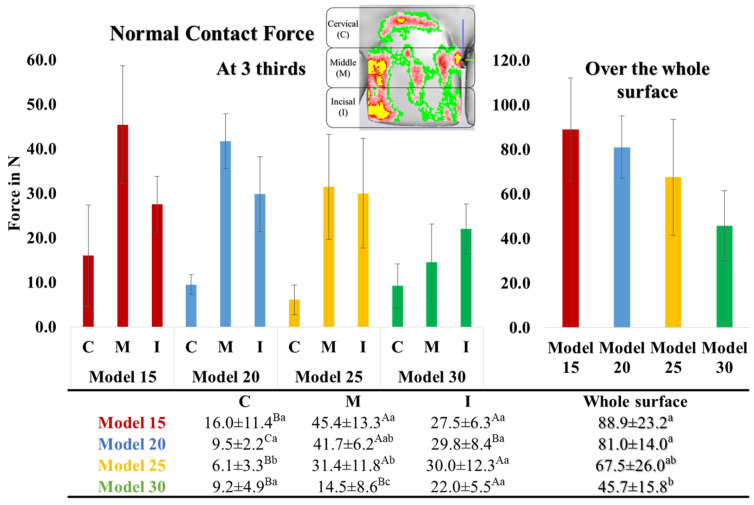
Normal contact force generated on Tooth 11 by aligners, made from Zendura FLX sheets thermoformed over models of different heights (15, 20, 25, 30 mm), and recorded using Fuji pressure-sensitive film. Different uppercase and lowercase superscript letters indicate a statistically significant difference within the same horizontal row and vertical column, respectively.

Figure 4 illustrates the local normal contact force across the entire facial surface of the crown of Tooth 11. The pressure-sensitive films recorded force values between 45.7 N and 88.9 N. Among the thermoformed aligners, those made over Model 15 exhibited the highest force, followed by Models 20 and 25, with no statistically significant differences between them. Aligners thermoformed over Model 30 showed the lowest contact force. The distribution of normal contact force was uneven, with certain areas experiencing higher forces than others. When the tooth surface was divided into three equal thirds, it was observed that the normal contact force at the cervical third (F_C_) was the lowest. The force at the middle third (F_M_) was significantly higher than the force at the incisal third (F_I_) and F_C_, except in the case of Model 30, in which F_I_ was the highest (Figure 4).

**Figure 5:**
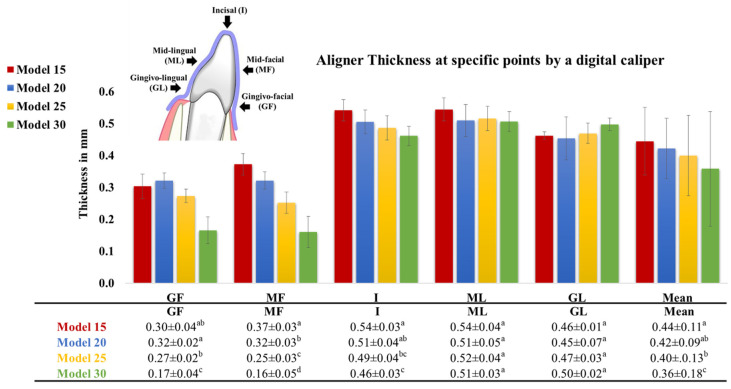
Thickness of aligners, made from Zendura FLX sheets thermoformed over models of different heights (15, 20, 25, 30 mm), recorded using a digital caliper at different points of the aligner splint covering the crown of Tooth 11. Different lowercase superscript letters indicate a statistically significant difference within the same vertical column.

**Figure 6:**
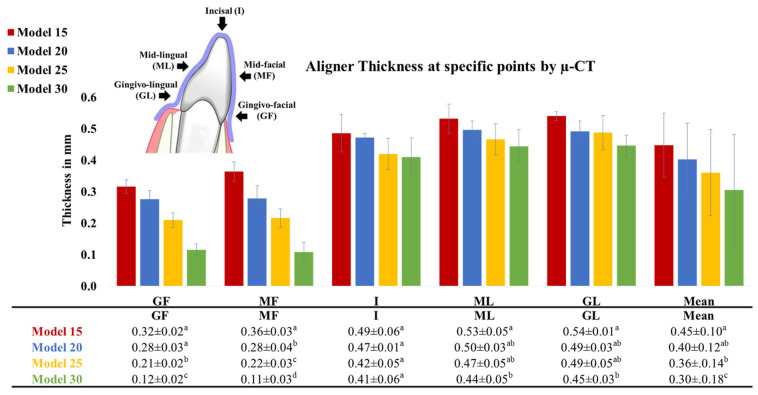
Thickness of aligners, made from Zendura FLX sheets thermoformed over models of different heights (15, 20, 25, 30 mm), recorded using a µ-CT device at different points of the aligner splint covering the crown of Tooth 11. Different lowercase superscript letters indicate a statistically significant difference within the same vertical column.

As depicted in Figure 5 and Figure 6, the thickness measurements obtained using the digital caliper and µ-CT were consistent and mutually validated. The initial thickness of the aligner sheets (0.75 mm) underwent a substantial reduction after thermoforming across the entire tooth surface, averaging between 0.3 and 0.5 mm (33–60% thinning). The least thinning occurred at the incisal edge (I) (0.4–0.5 mm; 30–50% thinning), while the most significant thinning was observed at the gingivo-facial part (GF) (0.1–0.3 mm; 60–85% thinning). Aligner thinning increased notably with increasing model height. There were noticeable differences in aligner thickness across various model heights on the facial surface; however, the differences in aligner thickness were less pronounced at the incisal tip and lingual surface.

## 5. Discussion

With the widespread utilization of orthodontic aligners, studying their biomechanics becomes crucial to address certain drawbacks and limitations associated with their use. Adopting a scientifically grounded approach in aligner manufacturing and application is essential for achieving optimal treatment outcomes. This study specifically explores the influence of model height, used in the thermoforming of aligner sheets, on both force transmission to Tooth 11 and aligner thickness.

Tooth 11 was chosen for this study due to its prevalence in experimental studies reporting forces. This allows for comparisons with other reports in the literature. Aligner treatment achieves incremental tooth movement through a series of aligner splints; each splint is capable of progressively repositioning the target teeth by approximately 0.2 mm for translations. The geometry of the aligner splint has a crucial role in determining the direction and extent of tooth movement. A predefined mismatch between the crown of the treated tooth and the splint generates force in three dimensions exerted on the contact surface between the aligner and the tooth [4].

The periodontal ligament (PDL) is a critical component of the dental structure, providing a cushion between the tooth and the bone. This ligament allows for the dissipation and absorption of forces exerted on the tooth during normal physiological activities, such as chewing or orthodontic adjustments. Without incorporating a model of the PDL in experimental setups, the forces measured do not accurately reflect the realistic, distributed nature of orthodontic force application and lead to the recording of force values that are significantly higher than the physiological orthodontic limit, which ranges from 0.1 to 1.2 N [17]. The force values obtained in this study (83.1–149.7 N) are significantly higher than those reported in the literature. This disparity may be attributed to the innovative methodology employed, which includes the use of pressure-sensitive films and a specialized scanner with software capable of directly quantifying force values. However, these pressure-sensitive films have limitations, as they provide localized one-dimensional force measurements on a single tooth surface. In contrast, clear aligners exert three-dimensional forces simultaneously across multiple tooth positions and directional axes, resulting in a lower overall resultant force. This discrepancy highlights a limitation of the current research approach. Nevertheless, these factors could be ignored, since all groups were evaluated and compared under the same standard conditions.

A clinical study by Barbagallo et al. [12] documented force values of approximately 28.0 N when using pressure-sensitive films. The presence of the PDL acts as a cushion that absorbs force, explaining the disparity between clinical and experimental outcomes. An experimental study by Cervinara et al. [13] reported pressure values around 3.3 MPa, which is comparable to the findings in this study, assuming that the force values result from the product of pressure (MPa) and area (mm^2^), and the measured area in the current study was around 40 mm^2^. Yet discrepancies observed across studies may be attributed to differences in material composition [5], trimming line design [10], the extent and type of tooth activation, and aligner thickness [8].

The inner surface of the aligner should closely contact the tooth surface to ensure the application of clinically effective force. However, in the present study, the force distribution demonstrated pressure and relief areas on the facial surface of the tooth. This resulted in variations in force levels across different points, aligning with observations from previous reports [10,13]. This uneven contact and force distribution may be attributed to the topography of the tooth surface, which can differ from one tooth to another.

Consistent with prior studies [10,16,18], the current outcomes showed significant material thinning (60–85%) after the thermoforming process, particularly at the gingival third [15,19], and less thinning at the incisal edge. Increased thinning of the aligner contributes to undesirable flexibility and weakening of the margins. Moreover, an increase in model height results in increased thinning of the thermoformed aligner, especially in the cervical areas. This leads to a significant reduction in transmitted force and a modification in force distribution, affecting both the magnitude and type of tooth movement. Principally, a 10% reduction in aligner material thickness could theoretically decrease exerted forces by up to 30%. This is because the moment of inertia is calculated using the third power of the material thickness in the direction of the acting forces [8,15].

Reducing the applied force on the tooth near the gingival area, closer to the center of resistance of the tooth, diminishes the likelihood of accomplishing bodily translation. Conversely, maintaining an appropriate aligner thickness at the gingival margins enhances stiffness, retention, and gingival adaptation. This, in turn, leads to improved force distribution and better control of tooth movement, allowing for greater force application at the gingival area, potentially enhancing control over bodily tooth movement [10,20,21]. To prevent additional weakening of the aligner margins, a straight extended trimming line was implemented instead of a scalloped design [10]. 

It is worth noting that this experimental study has certain limitations, including the investigation of only one type of aligner material (Zendura FLX, thermoplastic polyurethane-based multilayer sheets). Future studies may consider exploring different materials with varying compositions and thicknesses. Additionally, thickness values may vary depending on the types of teeth and the measurement locations [15]. Furthermore, factors such as material deformation during aligner placement and material swelling due to exposure to saliva were not considered.

Moreover, the actual tooth movement is determined by the system of forces acting on the tooth from all directions. When the tooth starts to move, it contacts the aligner on the palatal side, altering the resulting force system and tooth movement. However, in an in vitro study, the tooth is rigidly fixed to the maxilla and cannot move, effectively simulating only the initial contact between the aligner and the tooth. The force distribution suggests that different model heights lead to varying aligner thicknesses, resulting in different force distribution patterns. These patterns may influence the type of tooth movement, whether more “bodily” or “tipping”. We recognize the limitations of this in vitro study and understand that it is secondary to clinical evidence, and we adjust our recommendations accordingly.

To achieve desired and predictable outcomes, adjustments in aligner thickness throughout the treatment course are recommended. The use of 3D-printed aligners is also suggested to ensure a consistently uniform thickness across the entire surface and allow for intentional control over the aligner thickness [22]. The current methodology will be employed in subsequent studies to investigate the 3D-printed aligners.

## 6. Conclusions

The current in vitro study showed the following observations:

1. Thermoplastic aligner sheets experience substantial thinning by thermoforming.
2. Aligner thinning varies across the aligner splint covering the tooth, being minimal at the incisal part (30%) and maximal at the gingival section of the facial surface (85%).
3. Increased aligner thinning corresponds to lower force transmission.
4. Decreasing the model height diminishes the thinning effect, thereby preserving the thickness at the cervical part to enhance force application and promote bodily tooth movement.
5. The model height utilized for aligner thermoforming is recommended to be within the range of 15 mm from the occlusal table to the base.

Clinical Relevance: The present findings provide clinicians and aligner manufacturers with evidence-based guidance regarding the optimal model height for deep drawing aligner sheets. It is recommended that the model height for aligner thermoforming falls within the range of 15 mm. This ensures the maintenance of sufficient aligner thickness, thereby delivering adequate force for effective teeth movement.

## Data Availability

The datasets used and/or analyzed during the current study are available from the corresponding author on reasonable request due to privacy.
